# Genetic Transformation and Analysis of Rice *OsAPx2* Gene in *Medicago sativa*

**DOI:** 10.1371/journal.pone.0041233

**Published:** 2012-07-27

**Authors:** Qingjie Guan, Tetsuo Takano, Shenkui Liu

**Affiliations:** 1 Key Laboratory of Saline-alkali Vegetation Ecology Restoration in Oil Field (SAVER), Ministry of Education, Alkali Soil Natural Environmental Science Center (ASNESC), Northeast Forestry University, Harbin, China; 2 Asian Natural Environment Science Center (ANESC), The University of Tokyo, Nishitokyo City, Tokyo, Japan; TGen, United States of America

## Abstract

The *OsAPx2* gene from rice was cloned to produce *PBI121::OsAPx*2 dual-expression plants, of which expression level would be increasing under stressful conditions. The enzyme ascorbate peroxidase (APX) in the leaves and roots of the plants increased with increasing exposure time to different sodium chloride (NaCl) and hydrogen peroxide (H_2_O_2_)concentrations, as indicated by protein gel blot analysis. The increased enzyme yield improved the ability of the plants to resist the stress treatments. The *OsAPx*2 gene was localized in the cytoplasm of epidermal onion cells as indicated by the instantaneous expression of green fluorescence. An 80% regeneration rate was observed in *Medicago sativa* L. plants transformed with the *OsAPx*2 gene using *Agrobacterium tumefaciens*, as indicated by specific primer PCR. The *OsAPx*2 gene was expressed at the mRNA level and the individual *M. sativa* (T#1,T#2,T#5) were obtained through assaying the generation of positive T2 using RNA gel blot analysis. When the seeds of the wild type (WT) and the T2 (T#1,T#5) were incubated in culture containing MS with NaCl for 7 days, the results as shown of following: the root length of transgenic plant was longer than WT plants, the H_2_O_2_ content in roots of WT was more than of transgenic plants, the APX activity under stresses increased by 2.89 times compared with the WT, the malondialdehyde (MDA) content of the WT was higher than the transgenic plants, the leaves of the WT turned yellow, but those of the transgenic plants remained green and remained healthy. The chlorophyll content in the WT leaves was less than in the transgenic plants, after soaking in solutions of H_2_O_2_, sodium sulfite (Na_2_SO_3_), and sodium bicarbonate (NaHCO_3_). Therefore, the *OsAPx*2 gene overexpression in transgenic *M. sativa* improves the removal of H_2_O_2_ and the salt-resistance compared with WT plants. A novel strain of *M. sativa* carrying a salt-resistance gene was obtained.

## Introduction

Under drought and salt stress conditions, plants become dehydrated, which lowers the water potential of cells and organs. Furthermore, different physiologic processes are changed *in vivo* [1] and the APX (EC1.11.1.11) of the active oxygen detoxification system is activated. The plant antioxidant is an important aspect in the plant’s self-adaptive adjustment [2] The APX is the main enzyme for removing intracellular H_2_O_2_. APX has a high specificity and affinity for H_2_O_2_ and it catalyzes the reduction of H_2_O_2_ into water using reduced ascorbate acid as the reaction substrate. The reaction generates monodehydroascorbate which can be reduced to ascorbic acid using different methods [3], [4], [5]. APX is mainly localized in the cytoplasm, peroxisome, chloroplast, and mitochondria [6], [7], [8], [9]. These enzymes have the same function, but they play a discriminating role in the anti-oxidation of plants because of their different intracellular locations. APX provides cross-protection to the cytoplasm, thylakoid, and stromal mitochondria under light stress [10]. APX has a high affinity for H_2_O_2_, capturing H_2_O_2_ within many subcellular regions [11]. APX belongs to a multi-gene family [9]. *APx* gene overexpression improves enzyme activity for cleaning up hydrogen peroxide and it alleviates membrane lipid peroxidation injury caused by MDA [12]. At present, recombinant APX isozymes had been obtained *in vitro*. Some researchers had compared their structure and enzymatic kinetics, which revealed that they have consistent enzymatic properties and that APX isozymes have higher specificity for reduced ascorbic acid. However, APX isozymes are inactivated easily in the absence of substrate [13]. Different GSTs, such as *OsAPX*a and *OsAPX*b proteins for purification and fusion, have been expressed in prokaryotic cells by Lu et al. [14]. The comparative analysis of the enzyme kinetics showed that these proteins exhibited differences. The cytosolic APX of spinach leaves have significantly increased transcriptional levels under the same light and ultraviolet ray treatments. However, the chloroplast and peroxisome expression of APX is not affected [15]. Ma et al. [16] cloned the full-length complementary DNA (cDNA) of *Suaeda salsa* APX. RNA gel blot analysis showed that APX expression increases under salt stress and the enzyme activity also increases significantly, which indicated that the gene is induced by salt. APX was speculated to play a role in protecting *S. salsa* from oxidative damage caused by salt stress. Moving Arabidopsis from weak light to strong light induces the expression of two nuclear-encoded APX genes within 15 minutes. The expression was accompanied by a decrease in the ratio of reduced and oxidized glutathione [17]. Hong and Kao et al. [18] analyzed eight rice *OsAP*x genes using semi-quantitative PCR. The differences in the mRNA exhibited the dissimilarity between the *OsAPx*1 and the *OsAPx*2 gene under 150 mM NaCl stress. A study [19] showed that the transcriptional expression of the *OsAPx*2 rice gene was more rapid than that of the *OsAPx*1 gene under diseases stress. Lu et al. [20]. discovered that the root length and chlorophyll content of transgenic Arabidopsis plants overexpressing the *OsAPX*b gene was superior to those of plants overexpressing the *OsAPX*a gene in salt tolerance research. Differences in the salt and alkali tolerance of transgenic tobacco plants with over-expressed *OsAPx*1 and *OsAPx*2 genes have not been reported. *M. sativa* is an excellent pasture plant from the Leguminosae family that promotes nitrogen-fixing microorganisms. It is one of the most important crops in agriculture, which have an effect on soil improvement through nitrogen fixation. Furthermore, the areas cultivated with legumes constitute more than 3.2×10^7^ hectares worldwide [21].

**Table 1:** stress treatments of different oxidants.

oxidants	Concentration (mM)					
H_2_O_2_	0	1632	3263	4895	6527	8158
Na_2_SO_3_	0	5	10	20	30	40
NaHCO_3_	0	75	100	150	200	250

Salinization is a serious problem in soil management, it can decrease agricultural yield and ecological environment. Therefore, breeding different varieties of *M. sativa* with resistance plants is of particular significance to improving the characteristics of *M. sativa* and enlarging the arable land area. Currently, the resistance of *M. sativa* is improved by breeding new varieties via plant genetic engineering. Since the first *Agrobacterium-*mediated *M. sativa* translation experiment has been successful [22], heterogeneous gene in *M. sativa* has been studied gradually. The experiment involved the transfer of the *A. thaliana sAPX* gene into *M. sativa*, which resulted greater mRNA expression. The enzyme activity was improved by 3.8-fold and the reactive oxygen species produced by stresses was efficiently cleaned up [23]. The yield and survival of *M. sativa* treated with drought stress for three years was clearly improved with *Mn-SOD* cDNA [24]. *M. sativa* treated with Alfinl enhanced the expression of *MsPRP2* induced by saline, the salt tolerance, growth velocity [25]. Thus far, no experiment has reported on transgenic *M. sativa* about *OsAPx*2. In this paper the *OsAPx*2 gene was transferred into *M. sativa* cotyledon mediated by *A. tumefaciens* and the generation of T1 was assayed using specific primer PCR. The generation of T2 transcript levels was assayed using RNA blot analysis. Under salt stress, APX activity, physiologic and anti-oxidation indices in the recombinant *M. sativa* T2 generation were analyzed.

**Figure 1:**
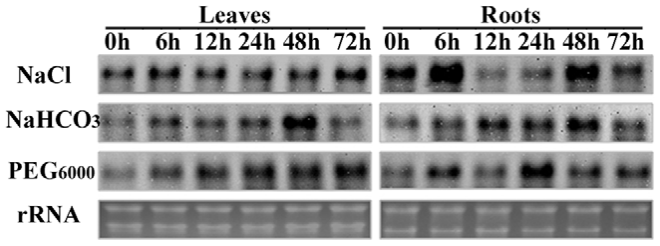
RNA gel blot analysis of *OsAPx2* gene expression under different stresses. RNA gel blot analysis of *OsAPx2* gene expression in rice roots and leaves treated with 100 mM NaCl, 20 mM NaHCO_3_, and 10% PEG 6000 for 0, 6, 12, 24, 48, and 72 h. 10 µg sample volume of total RNA.

## Materials and Methods

### Chemicals

DIG-labelled and CDP-StarTM were purchased from Amersham Pharmacia. Trizol was purchased from Invitrogen. 3,3′-diaminobenzidine staining (DAB) was purchased from Sigma. The other chemicals are AR grade.

**Figure 2:**
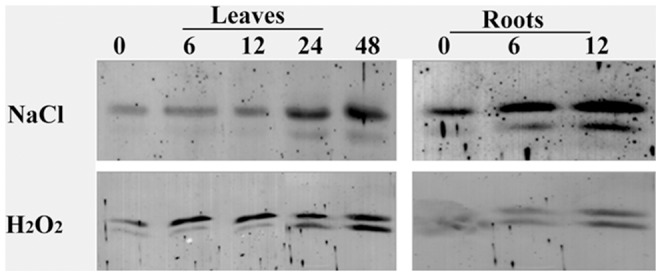
Protein gel blot analysis of *OsAPx*2 expression in the leaves and roots of rice under NaCl and H_2_O_2_ stress. The leafs were treated for 0,6,12,24,48 h respectively and the roots were treated for 0,6,12 h respectively.

### Plant Material

The *M. sativa* seeds were obtained from the Academy of Agricultural Sciences of Changchun (Jilin Province, China). The *A. tumefaciens* (EHA105 strain) containing the dual-expression plasmid *PBI121*-*OsAPx*2 was obtained. The probe with *OsAPx*2 was marked by Domain Information Groper (DIG) from the library.

**Figure 3:**
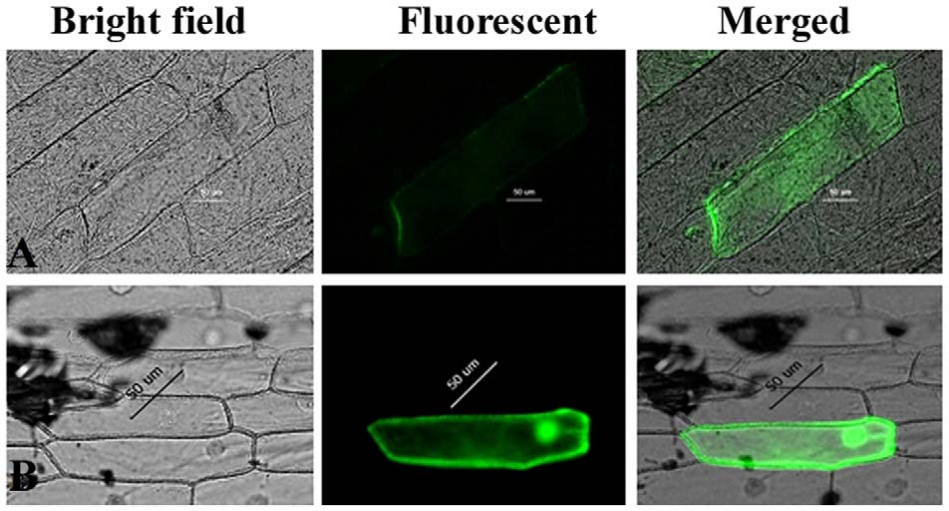
Subcellular localization of GFP fusion protein in the onion epidermis instant expression system. A: Localization of pBI121-*OsAPx2*::GFP protein in the cytoplasm in the micrographs; B: pBI121-GFP proteins were located throughout the cytoplasm.

Rice seedlings at the three-leaf stage (two weeks) were used in the subsequent stress tolerance assays. The plants were treated with different stresses, namely, 100 mM NaCl, 60 mM NaHCO_3_, and 10% (w/v) polyethylene glycol 6000(PEG 6000) for 6, 12, 24, 48, and 72 h. Plants treated with water were used as the controls. All treatments were performed in triplicate. The roots and leaves of rice seedlings were harvested, immediately frozen in liquid nitrogen, and stored in a freezer at −80°C to be used for testing.

**Figure 4:**
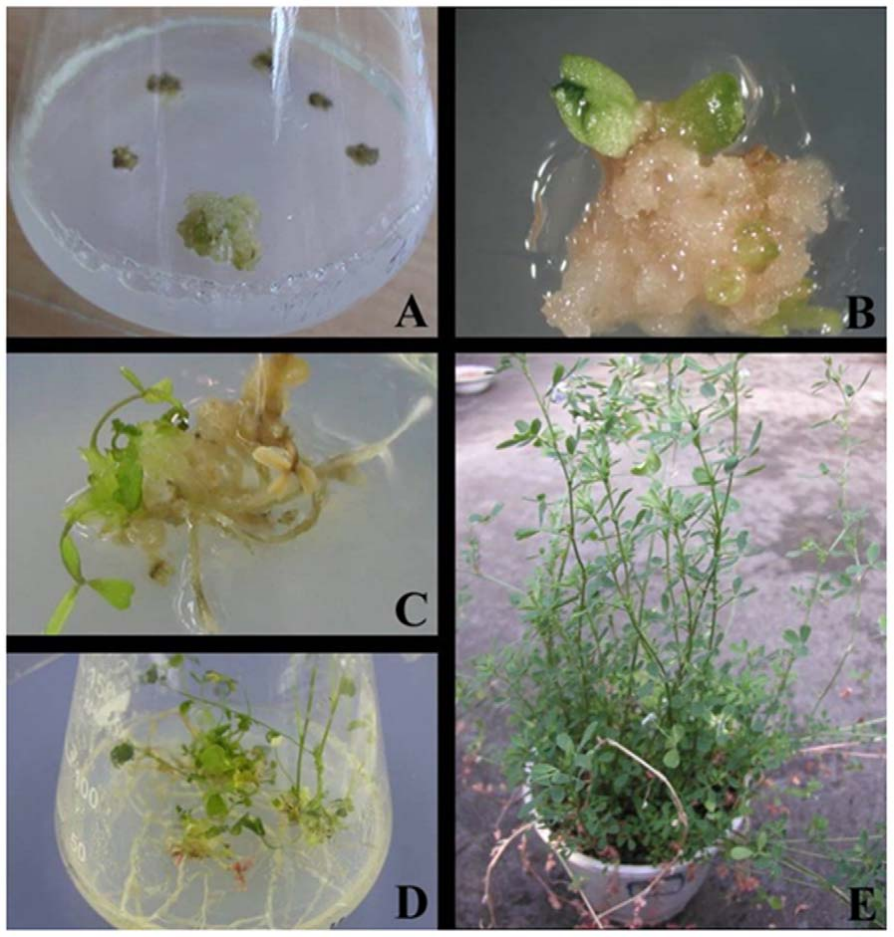
Procedure for obtaining *M. sativa* carrying the *OsAPx2* gene. A: The kanamycin-resistant callus, B: The kanamycin-resistant embryo, C: The kanamycin-resistant bud of resistant kana, D: the transgenic youth plant with *OsAPx2*, E: The transgenic plant with *OsAPx2*.

### Culture Medium

The callus induction medium used was Murashige and Skoog (MS) medium containing the following: 2 mg/L 2,4-dichlorophenoxyacetic acid, 1 mg/L benzylaminopurine (6-BA), 8 g/L agar, and 30 g/L sucrose. The screening culture medium for bud differentiation contained MS with 2 mg/L kinetin, 0.15 mg/L 6-BA, 0.3 mg/L α-naphthalene acetic acid, 50 mg/L kanamycin, 500 mg/L carbenicillin, 100 µM acetosyringone, 8 g/L agar, and 30 g/L sucrose. The culture medium for promoting root growth contained 1/2 MS and 0.5 g/L yeast extract.

**Figure 5:**
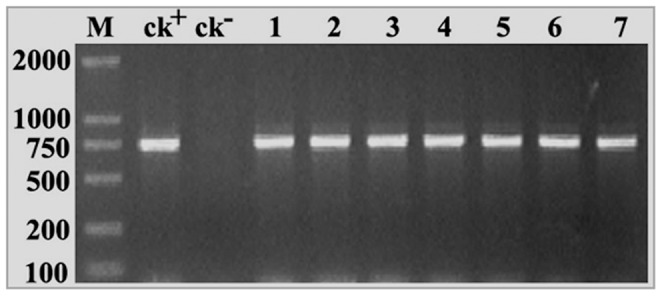
PCR identification of transgenic alfalfa with rice *OsAPx2*. CK+: pBI 121*-OsAPx2* plasmid, CK-: untransformed alfalfa, 1–7: transgenic alfalfa with rice *OsAPx2*. The volume was diluted up to100 then measured in a pipette with 1 µL for the PCR template. The result shows that 48 amplification products by PCR are identified with pBI121*-OsAPx2*’s (750 bp), and none of the products was identified with negative blank (ck-).

## Methods

### Enzymatic and mRNA Transcription Expression Analysis of the *OsAPx*2 Gene Under Salt Stress

The specific primers for the gene probe were designed based on the GenBank login gene *OsAPx*2 (AB053297). The variance probe was signed with DIG. (*OsAPx*2. P1∶5′-aagcctttagagagcggga-3′, P2∶5′-gcttggtaactttgaaactcc-3′) 5 µg of RNA was used in the denature electrophoresis, membrane transfer, variance probe hybridization, membrane cleaning, and signal detection by CDP-Star [26].

**Figure 6:**
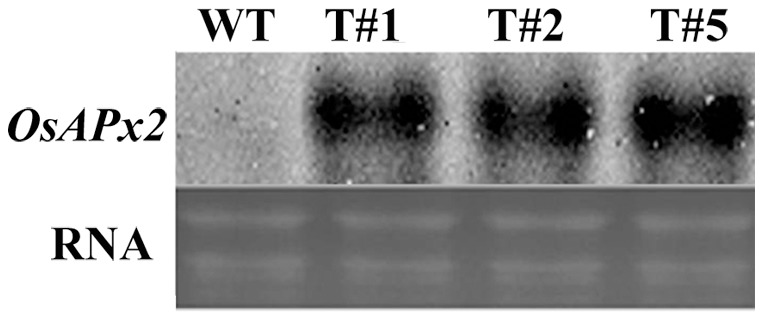
RNA gel blot analysis on T2 transgenic alfalfa expressing *OsAPx2*. WT: untransferred alfalfa #1, 2, and 5:T2 transferred alfalfa with *OsAPx2.*

The quality assay was performed on the plant protein via the Bradford method [27]. The materials were treated as follows: 500 mg of the finely cut leaves and roots were weighed, and then soaked in NaCl and H_2_O_2_, respectively. At the same time, an equal amount of sample was soaked in water as the control. The proteins were extracted using phosphate-buffered saline (PBS) contain 5 mM Ethylenediaminetetraacetic acid disodium salt (EDTANa_2_), 0.001% Phenylmethanesulfonyl fluoride (PMSF), pH 8.0. The materials were rubbed rapidly in Potter-Elvehjem Tissue Grinders under ice-cold conditions, and the supernatant liquid was collected after filtration at 4°C and 12000 rpm for 20 min in a 1.5 mL tube. A similar volume of crude protein was extracted for the sodium dodecyl sulfate polyacrylamide gel electrophoresis(SDS-PAGE). The protein was transferred onto a pyroxylin membrane using semi-electric transfer method instrument [28]. The expressed *OsAPx*2 under stresses was assayed using primary anti-*OsAPx*2 polyclonal antibodies. Secondary rabbit anti-goat antibodies AP-labeled were used for protein blot analysis with NBT as the substrate (Kit supplied by the Tiangen Corporation).

**Figure 7:**
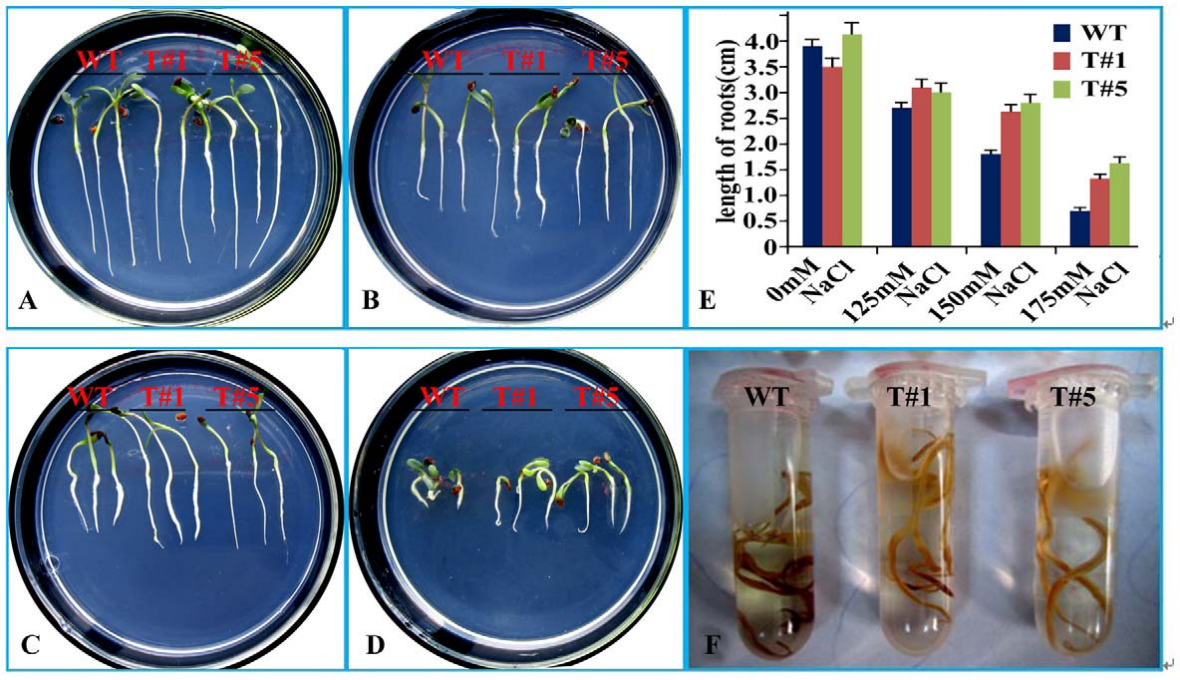
Relative salt tolerance of wild type (WT) and transgenic plants with *OsAPx2* overexpression in *Medicago sativa* (T#1 and T#5) under salt stress. A: WT and transgenic seeds germinated in 1/2 ·MS solid medium agar plates for 1 week. B, C, D: WT and transgenic seeds germinated in 1/2 MS with 125, 150, and 175 mM NaCl. E: Effects of salt on the root lengths of the WT and transgenic plants overexpressing the *OsAPx2* gene. The figure shows the root lengths of the WT and two transgenic lines (T#1 and T#5) after 1 week of salt treatment. This shows that the root lengths of the transgenic plants are longer than those of the WT plants. Data on the profile E note mean ±SD (n = 20). F: Analysis of the content of H_2_O_2_ via DAB shows that the WT has higher content than the transgenic type.

**Figure 8:**
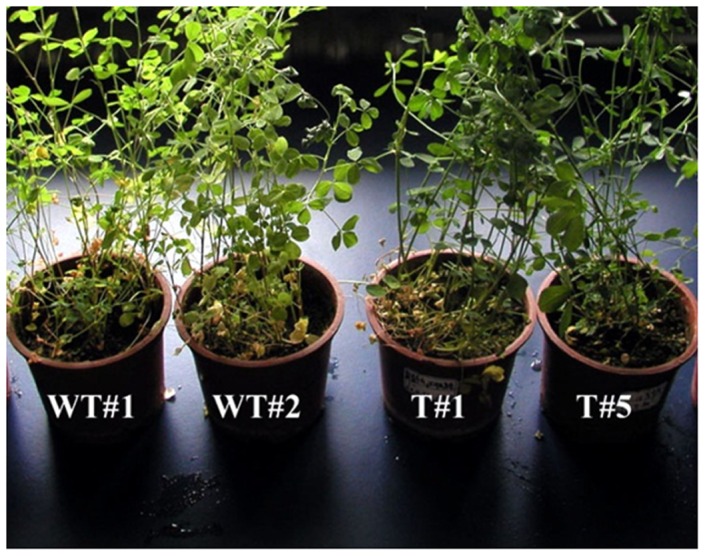
The growth status of the WT and T2 plants under NaCl stress. WT = wild type; T2 = the first generation carried *OsAPx2*, the root of the adult plants were soaked in 200 mMNaCl solution and the plants were maintained in natural light for 7 days.

### Subcellular Localization of OsAPx2 Genes

The subcellular location of *OsAPx*2 gene expression was predicted based on its amino acid sequence in NCBI (http://www.ncbi.nlm.nih.gov/). To further verify the subcellular location, a *Kpn* I site was designed before the 5′-end ATG of the *OsAPx*2 genes for synthesizing upstream primer: *OsAPx*2 (P3∶5′-ggatccatgggcagcaagtc-3′); An *Spe* I site was designed after the 3′-end for synthesizing downstream primer: (P4∶5′-actagtttcctcagcaaatcc-3′), and then the primers were cloned into a pMD18-T vector. If the sequence was correct, pMD18-T*::OsAPx*2 and *PBT121-35SMCS::GFP* were hydrolyzed by *Kpn* I and *Spe* I. Destination DNA was recycled for connecting, transforming, and constructing *PBI121::OsAPx2::GFP* expression plasmids. The building plasmids were introduced into onion epidermal cells via gene gun transformation. The green fluorescence, which demonstrated the expression location, was observed by a confocal laser scanning microscope (Olympus).

**Table 2:** APX activity AVONA.

source of variation	SS	freedom	MS	F value	p-value
**Interblock**	0.1371	2	0.0686	12.186	0.0077
**Within treatment**	0.0338	6	0.0056		
**Total variation**	0.1709	8			

**Table 3:** result of APX activity multiple comparison by Duncan’s method.

treatments	mean	Significance level
T#1	0.4095	a
T#5	0.3896	a
WT	0.1383	b

Note: a and b in this table indicate variation significance at α = 0.05 level(the same below).

### Heredity Transformation

The method was performed based on the study of Jin et al. [29].

**Table 4:** MDA content multiple comparison by Duncan’s method.

MDA(µmol/g)	Treatment time(h)				
	0	12	24	48	72
WT	2.0838^a^	2.3015^a^	3.3893^a^	6.8098^a^	11.632^a^
T#1	2.0586^a^	2.0867^b^	2.5194^b^	3.8703^b^	4.3147^b^
T#5	1.9854^a^	2.0551^b^	2.4791^b^	3.7678^b^	3.9883^b^

### Molecular Examination

#### PCR assay

Genomic DNA from the recombinant and wild-type (WT) plants of T_1_ generation was diluted 100-fold. Then, 1 µL was withdrawn for PCR template. Both upstream and downstream primers were designed based on the ORF sequences of *OsAPx*2 (AB053297) as follows: P5∶5′-gagcatgggcagcaagtcgt-3′: P6∶5′-cttattcctcagcaaatccc-3′, which were synthesized by Shanghai Sangon Biological Engineering Technology & Services Co., Ltd. The agarose gel electrophoresis carried out for 45 minutes with 1× TAE buffer contains 0.8%(w/v) agarose. Pictures of the samples were taken using VILBER LOURMAT.

**Figure 9:**
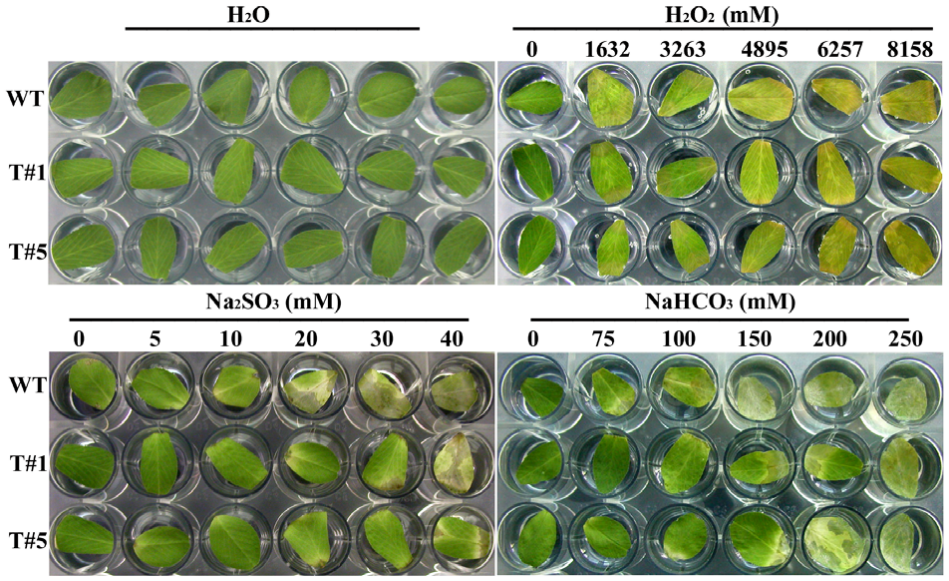
Antioxidative activity in the leaves of transgenic and WT alfalfa under different salinity levels.

#### RNA gel blot assay

The total RNA was obtained using Trizol. Denaturing gel electrophoresis was carried out to examine the transformation of *OsAPx2* gene, which was marked with an probe in the DIG with RNA gel blot analysis, as referred from Sambrook et al. [26].

### Anti-salinity Analysis

The resistant *OsAPx2* overexpression under salt stress was analyzed to study the positive generation of T2 under Promoter 35S from Cauliflower Mosaic (*CaMV35S*). The seeds of the WT and transgenic *OsAPx*2 (T#1, T#5) were sown in 1/2 MS culture medium containing 0, 125, 150, and 175 mM NaCl for 7 days. Then, the effects of *OsAPx*2 overexpression on the ability to resist salt stress were analyzed in detail.

The Hydrogen Peroxide Content was Assayed through DAB According to Hückelhoven [30].

The plants (WT, T#1, and T#5) were cultured at 24°C ±2°C with illumination for 10 h at a light intensity of 30 µmol·m^−2^·s^−1^ to 40 µmol·m^−2^·s^−1^ after soaking in 200 mM NaCl. The enzyme properties were assayed using the following: The total enzyme activity of APX [3]; the content of MDA [31] at 0, 12, 24, 48, and 72 h, respectively. The enzyme solution contained 0.5 mM l-ascorbic acid and 50 mM phosphate equilibrated with 0.2 mM H_2_O_2_ buffer (pH 7.0) with suitable crude enzyme extract.

### Analysis of Anti-oxidation Injury in Transgenic OsAPx2 Plant Leaves

To verify if the plant overexpressing *OsAPx*2 is resistant to stress, their ability to scavenge the H_2_O_2_ produced during stress and the ability to resist anti-oxidation injury were compared. The leaves of the WT and transgenic plants were soaked in solutions with different concentrations, as shown Table1.

### Data Analysis

Analysis of variance (AVONA) and multiple comparison by software data processing system(DPS) (version 7.05).

## Results and Analysis

### Expression and Analysis of OsAPx2 Genes in Rice Under Stress

Total RNA was extracted from the root and leaf samples. Then, the total RNA concentration was determined using a ultraviolet-visible spectrophotometer. Up to 10 µg of each sample was prepared for the formaldehyde denatured electrophoresis, membrane transfer, and DIG variance probe hybridization. RNA gel blot analysis showed that the induction of gene expression (Figure. 1).

With prolonged exposure to the NaCl, NaHCO_3_, and PEG 6000 stress treatments, *OsAPx*2 expression increased at the transcriptional level. The gene expression with the NaCl solution was significantly increased after 6 h, which is likely close related to salt and alkali stress.

### Analysis of OsAPX2 Protein Expression Under Stress

The results of the protein gel blot analysis are shown in Figure 2. The signals of the leaf sample under both 100 mM NaCl and 3263 mM H_2_O_2_ were more distinct with time, by which the amount of APX2 increased gradually. The APX2 signal in the root sample also increased gradually under 100 mM NaCl and 3263 mM H_2_O_2_ with time. The salt stress likely stimulated the APX2 expression. More H_2_O_2_ was produced with prolonged exposure to the salt stress [32]. The H_2_O_2_ is converted into water by the APX isoenzyme under normal physiologic conditions [9]. Increasing the amount of APX2 reduces the injury caused by the H_2_O_2_ accumulation. Conversely, the increased H_2_O_2_ concentration increases the amount of APX2. Therefore, *OsAPx*2 overexpression may improve the ability of plants resisting salt stress.

### Subcellular Localization of OsAPx2::GFP in Onion Epidermal Cells

*OsAPx*2 genes were predicted to be localized in the cytoplasm with a reliability index of 0.45 (http://www.ncbi.nlm.nih.gov/).

*PBI121::OsAPx*2*::GFP* plasmid DNA was constructed under the control of the CaMV35S promoter. The plasmid DNA was wrapped with aurum powder to replace arginine and CaCl_2_. The plasmid DNA was introduced into onion epidermal cells using a gene gun, and cultured in the dark for 18 h to 24 h. The onion epidermal cells were torn and squashed. GFP fluorescence was observed using a confocal laser scanning microscope. The results of subcellular localization analysis revealed that the *OsAPx*2*::GFP* fusion protein showed green fluorescence in the cytoplasm (Figure 3-A) and the control GFP protein in the cytoplasm and the whole cells were green (Figure 3-B), which indicated the cytoplasmic localization of the *OsAPx*2 gene. The results coincided with the results predicted from the network information software.

### Rice OsAPx2 Gene Genetic Transformation

The white-green callus was transformed by *A.tumefaciens* EHA105 performed in the dual-expression plasmid *PBI121*-*OsAPx*2. Up to 60 samples carrying the *OsAPx*2 gene were obtained through the tissue culture technique (Figure 4.). The callus was incubated in three days. Then, the differentiation screening was performed on the regeneration bud in the medium for three weeks. The non-kanamycin-resistant callus turned brown and eventually died. Kanamycin, may have caused division in the cell mass (Figure 4-A as indicated by arrows). After five weeks, the embryo emerged with kanamycin-resistant cells (Figure 4-B as indicated by an arrow). Then the regenerating bud carrying the *OsAPx*2 gene was be obtained (Figure 4-C). After which, the transgenic regenerating bud was transferred into the medium for improving root growth for four weeks (Figure 4-D).

### PCR Assay

The *OsAPx*2 gene was transformed into *M. sativa* callus by means of genetic transformation, 60 recombinants were obtained through Kanamycin resistance differentiation and screening, all of them and WT samples were PCR analysis. 48 samples contained *OsAPx*2 gene, which were identified with the same segment of plasmid *pBI121*-*OsAPx*2 (750 bp) however, WT (CK^-^) was not seen in the band (proportion results of PCR assay was shown as Figure 5.). Thus the *OsAPx*2 gene was transferred successfully into the 48 individual samples and the transformation ratio reached up to 80%. The T1 generation seeds were obtained.

### RNA Gel Blot Analysis

The total RNA of T2 positive plants and the blanks were extracted by Trizol Probes. The RNA gel blot analysis results were shown as Figure 6. The control samples had no signals but the transgenic plants all expressed the gene, which showed that the rice *OsAPx2* gene has been transformed successfully. The heterogeneous genes were transcribed and expressed, and the new *M. sativa* lines carrying the rice *OsAPx*2 gene were obtained.

### Salt Resistance Analysis

The root lengths under the 7-day stress with different NaCl concentrations were shown in Figure 7. The root lengths of the WT plants were affected significantly by the 150 mM and 175 mM NaCl treatments.This shows that the root lengths of the transgenic plants were longer than those of the WT plant**s (** Figure 7-A,B,C,D,E**)**. However, the roots were unaffected by the 125 mM NaCl treatment. Analysis of theH_2_O_2_ content via DAB showed that WT had higher content than transgenic plants(Figure 7-F). Therefore, *OsAPx*2 gene overexpression removed the H_2_O_2_ produced during NaCl stress, and reduced plant injury.

The salt resistance was tested by NaCl treatments with the same duration. The results were shown in Fig. 8, which demonstrated that the WT plants were chlorotic, whereas the T2 plants looked healthy.

### APX Activity Assay

The APX activity of WT and transgenic plants were assayed and analyzed as shown in Table 2. The multiple comparison analysis as shown in table 3.With the CaMV35S promoter, the *OsAPx*2. gene was overexpressed, and the APX activity was clearly improved. The APX activity of T#1 and T#5 were more than WT plants. By means of multiple comparison of Duncan’s method, which showed there was a significant variation between T(T#1,T#5) and WT, but it was insignificant between T#1 and T#5 plants.

### Assay Content of MDA

MDA, which causes membrane lipid peroxidation, is a final product of the accumulation of reactive oxygen species under salt stress [33]. against the stress. The MDA content under the different NaCl treatments were shown in Table 4.

The MDA content in cells under stress can directly indicate the resistance of the plant. In all treatments, the MDA content increased with exposure time to the NaCl stress. After treating 24 h, the MDA content of the WT was higher than that of the transgenic plants. This indicated that the presence of the *OsAPx*2 gene can inhibit or eliminate the accumulation of reactive oxygen species in the cell. There was not significant variation of MDA content between the T#1 and T#5 treatments, but there was a significant variation between the WT and the other treatments after 12 h in stress.

### Comparative Analysis of Leaf Anti-oxidation

The leaves of the WT and the transgenic plants were soaked in solutions containing H_2_O_2_, Na_2_SO_3_, NaHCO_3_, and water (control). The results were shown in Figure 9. Based on the results, the *OsAPx*2 gene improved the capacity of alfalfa to eliminate reactive oxygen species, thereby increasing the salt resistance of the plants.

In the 18-hour treatment, the differences in stress resistance were significant among the different treatments. The injury to the leaves increased with increasing salt concentrations and they gradually turned brown. The WT leaves turned yellow faster than the transgenic leaves under the 4895 mM H_2_O_2_. When the NaHCO_3_ and Na_2_SO_3_ concentrations reached 150 mM and 20 mM, respectively, the WT leaves experienced more injury than the transgenic leaves, and the chlorophyll content was evidently decreased.

## Discussion

Reactive oxygen species accumulated at high levels in plant cells during salt stress. The proteins, membrane lipids, and other cell components are reduced, experiencing serious injury. However, the levels of reactive oxygen species in plant cells can reach equilibrium through enzyme and non-enzyme systems under normal conditions [34], [35]. The rice *OsAPx* gene belongs to a multi-gene family. RNA gel blot analysis showed that the transcription of the *OsAPx*2 gene increased significantly with time of exposure to NaCl, NaHCO_3_, and PEG 6000. This result coincides with the results of Székely et al. [36], Nounjan et al. [37], and Chen et al. [38]. The yield of APX2 enzyme increased gradually under the NaCl and H_2_O_2_ stress, as indicated by the protein gel blot analysis [32]. Longer stress time promoted the accumulation of hydrogen peroxide. The H_2_O_2_ is converted into water by APX isozyme under normal physiologic conditions [39]. Increasing the amount of APX2 reduces the injury caused by H_2_O_2_ accumulation. Conversely, increasing H_2_O_2_ concentrations increases the amount of APX2 such that *OsAPx*2 overexpression likely improves the ability to resistant salt.

Currently, plant genetic engineering is one of the most important ways for in improving the resistance of *M. sativa*. *Agrobacterium-*mediated transfection has greatly improved the study of transgenic crops [40]. In 1986, Deak et al. [41] obtained progenitive resistant samples.

After the first successful *Agrobacterium*-mediated transformation [42], more studies were performed on the subject. Furthermore, the seedling are not infected by the bacterium and harmed by the antibiotics [43].

In this paper, the T2 generation overexpressing the *OsAPx*2 gene was more resistance than the WT under the 200 mM NaCl stress. The regenerated plants were obtained using calli from organ transgenic *OsAPx*2 *M. sativa*. The transformation rate was up to 80% under specific primer PCR. *OsAPx*2 was overexpressed in the generation T2 as indicated by RNA gel blot analysis. Under NaCl stress, the potential for budding of the transgenic plants was higher than the WT. The H_2_O_2_ content of the transgenic plants was lower than that of the WT under DAB analysis. The transgenic plants were more resistant as indicated by the 200 mM NaCl using a pot test. The APX activity of the transgenic plants was 2.89-fold that in the WT [44]. This is also achieved when the CaMV35S was combined with the *OsAPx*2 gene, which improved enzyme production. Therefore, the plant carrying the *OsAPx*2 gene can improve the ability to detoxify reactive oxygen species. *OsAPx*2 gene overexpression promotes the removal of hydrogen peroxide [45]. Plant resistance to salt depends on the stability of the cell membrane, or the ability to maintain the integrity of the system, ion selective absorption, and other functions [46]. MDA, the finally product of membrane lipid peroxidation caused by accumulating reactive oxygen species, cross-links lipids, nucleic acid, saccharides, and proteins. MDA reacts strongly with many types of materials in the cell, which reduces the available unsaturated fatty acids, which changes the lipid membranous structures and functions. It can cause a series of changes in the physiology and metabolism [47], [48].

The MDA content in the WT and transgenic *M. sativa* was greater than that before the 48-hour and 72-hour treatments with the different NaCl concentrations. However, the content was lower in the transgenic than the WT plants. The reduction of accumulated reactive oxygen species in the cell during stress is caused by the *OsAPx*2 overexpression in *M. sativa*. The membrane lipid peroxidation in the cell is observed as leaf chlorosis. SOD overexpression in the chloroplast of transgenic *M. sativa* improves their tolerance to oxidative stress and freeze injury [49]. The leaves were soaked in different concentrations of H_2_O_2_, Na_2_SO_3_, and NaHCO_3_, indicated that the transgenic plants were significantly more tolerant of oxidative stress than the WT. *OsAPx*2 overexpression improves the detoxification of reactive oxygen species and reduces membrane lipid peroxidation. The gene incorporated into *M. sativa* is of great significance to the development of Chinese graziery.
